# Pharmacokinetics and pharmacodynamics of isopropoxy benzene guanidine against *Clostridium perfringens* in an intestinal infection model

**DOI:** 10.3389/fvets.2022.1004248

**Published:** 2022-09-29

**Authors:** Yixing Lu, Liuye Yang, Wanying Zhang, Jie Li, Xianfeng Peng, Zonghua Qin, Zhenling Zeng, Dongping Zeng

**Affiliations:** ^1^Guangdong Provincial Key Laboratory of Veterinary Pharmaceutics Development and Safety Evaluation, National Risk Assessment Laboratory for Antimicrobial Resistance of Animal Original Bacteria, College of Veterinary Medicine, South China Agricultural University, Guangzhou, China; ^2^Guangdong Laboratory for Lingnan Modern Agriculture, Guangzhou, China; ^3^Guangzhou Insighter Biotechnology Co., Ltd., Guangzhou, China

**Keywords:** isopropoxy benzene guanidine, pharmacokinetic/pharmacodynamic (PK/PD), intestinal infection model, *Clostridium perfringens*, broiler

## Abstract

This study aimed to evaluate the antibacterial activity of isopropoxy benzene guanidine (IBG) against *C. perfringens* based on pharmacokinetics/pharmacodynamics (PK/PD) modeling in broilers. The PK parameters of IBG in the plasma and ileal content of *C. perfringens*-infected broilers following oral administration at 2, 30, and 60 mg/kg body weight were investigated. *in vivo* PD studies were conducted over oral administration ranging from 2 to 60 mg/kg and repeated every 12 h for 3 days. The inhibitory *I*_*max*_ model was used for PK/PD modeling. Results showed that the MIC of IBG against *C. perfringens* was 0.5–32 mg/L. After oral administration of IBG, the peak concentration (*C*_*max*_), maximum concentration time (*T*_*max*_), and area under the concentration-time curve (AUC) in ileal content of broilers were 10.97–1,036.64 mg/L, 2.39–4.27 h, and 38.31–4,266.77 mg·h/L, respectively. After integrating the PK and PD data, the AUC_0 − 24*h*_/MIC ratios needed for the bacteriostasis, bactericidal activity, and bacterial eradication were 4.00, 240.74, and 476.98 h, respectively. For dosage calculation, a dosage regimen of 12.98 mg/kg repeated every 12 h for 3 days was be therapeutically effective in broilers against *C. perfringens* with MIC ≤ 2 mg/L. In addition, IBG showed potent activity against *C. perfringens*, which may be responsible for cell membrane destruction. These results can facilitate the evaluation of the use of IBG in the treatment of intestinal diseases in broilers caused by *C. perfringens*.

## Introduction

Necrotizing enteritis (NE) is widely spread in broilers, which poses a major economic burden on poultry industry worldwide (1). NE was first described by Parish (2), with high morbidity and mortality (3). The pathogen of NE is *Clostridium perfringens* (*C. perfringens*), a Gram-positive spore-forming anaerobic bacteria (4). Toxins produced by *C. perfringens* can cause gastroenteritis, enterocolitis or enterotoxaemia in humans and animals (5). The use of antibiotic growth promoters in livestock industry must be decreased worldwide to delay the spread of antibiotic resistance (6, 7). However, these measures lead to the high prevalence of NE (8). Broilers are prone to NE at 2–6 weeks of age (9). Antibiotic therapy can effectively control NE. Drug resistance of *Clostridium perfringens* clinical isolates is becoming common because of the frequent use of antibiotics (10). Thus, developing new drugs different from existing drugs is an effective method to overcome antibiotic resistance.

Guanidine compounds have been widely used in the treatment of various diseases because of their biological activities, and they are potential candidates for structural modification of new drugs (11–13). Liu et al. reported that metformin, an antidiabetic drug, promotes intracellular accumulation of doxycycline to restore antibiotic activity against multidrug-resistant bacteria (14). Pi et al. reported that robenidine analog NCL195 alone or in combination with EDTA, polymyxin B non-apeptide, and polymyxin B has good antibacterial activity against various bacteria including *Staphylococcus aureus* (15). As a new candidate for substituted guanidine compounds, isopropoxy benzene guanidine (IBG) has been proven to be effective against Gram-positive bacteria (16, 17). IBG disrupts the cell membranes of drug-resistant *Enterococci* and *Staphylococcus aureus*. In addition, IBG can affect colistin against colistin-resistant *Salmonella* (18). IBG supplementation effectively improves the average daily gain and reduces diarrhea rate of broilers without adverse reactions (19).

The present study sought to determine the pharmacokinetic (PK) data of IBG in plasma and ileal content. The PK/pharmacodynamics (PD) indexes required for different levels of antibacterial effectiveness by using the inhibitory *I*_*max*_ model were also analyzed. Furthermore, the formulation of the dosage regimen of IBG in broilers could be used to formulate a reasonable dosage for treating NE.

## Materials and methods

### Antibiotic and bacteria

Isopropoxy benzene guanidine (99.9%) was provided by Guangzhou Insighter Biotechnology (Guangzhou, China). Mueller–Hinton broth and Mueller–Hinton agar were obtained from Qingdao Hope Bio-Technology Co., Ltd. (Qingdao, China). Tryptone-sulfite-cycloserine agar was obtained from Guangdong Huankai Microbial Technology (Guangdong, China). Twenty-four isolates of *C. perfringens* were used, including a standard strain (ATCC13124) was purchased from the Chinese Veterinary Culture Collection Center and 23 strains isolated from broilers in five cities in Guangdong province from March to November in 2021.

### Animals

Two-week-old healthy Sanhuang broilers with weights 100 ± 10 g were used in this study. Broilers were allowed 7-day acclimation prior to experiments. All broilers were allowed with antibiotic-free food and water supply *ad libitum*. All procedures were approved by the Institutional Animal Care and Use Committee of South China Agricultural University (Approval Number: 2022A001).

### Determination of MIC, MBC, MPC, and PAE

The susceptibility of the selected *C. perfringens* isolates to IBG in MH broth was evaluated in accordance with the micro-dilution method recommended by the CLSI (20). Minimal inhibitory concentration (MIC) was defined as the lowest concentration of IBG that inhibited the visible bacterial growth after 24 h of incubation. The MIC in ileal content was also evaluated in using the micro-dilution method (21). The mutant prevention concentration (MPC) of IBG was determined using the agar method (22). The 10^10^ CFU/mL *C. perfringens* strains were inoculated on the agar plates containing serial concentration of IBG (1 MIC, 2 MIC, 4 MIC, 8 MIC, 16 MIC, and 32 MIC) and cultured at 37°C for 72 h. The MPC was defined as the lowest concentration of IBG on agar plates without bacterial growth.

For the post-antibiotic effect (PAE) determination, the bacterial was exposed to three different concentrations (1 MIC, 2 MIC, and 4 MIC) of IBG for 1 or 2 h. The media containing IBG was removed by centrifuge at 12,000 × g for 5 min. The bacterial was re-grew in fresh media without IBG for another 24 h. The bacterial numbers were determined at different time points. The PAE was the time difference (in hours) for antimicrobial-treated bacterial to increase in number by 1 log10 minus the same determination for non-treated cultures of the same test bacterial (23).

### *In vitro* time-killing curves

Different concentrations of IBG: 1/4MIC, 1/2MIC, 1MIC, 2MIC, and 4MIC were prepared in MH broth, the tubes were then inoculated with *C. perfringens* (10^6^ CFU/mL) and incubated at 37°C. The bacterial count (CFU/mL) was determined for each tube after 0, 1, 2, 4, 6, 8, 12, and 24 h of incubation. In brief, 100 μL of culture was obtained for each time point, and serially diluted, and the colonies were counted the next morning. The limit of detection (LOD) was 10 CFU/mL. All experiments were performed in triplicate.

### Establishing *C. perfringens* infection model

Based on references and proper modification (24, 25), broilers were infested by oral challenging with coccidial sporulated oocysts propagated from field isolates (30,000/in 1 mL/bird). After 4 days, broilers oral gauge with 1 mL of culture containing 10^9^ CFU/mL of *C. perfringens* ATCC13124 for 3 days. Broilers were observed after inoculation for clinical symptoms and pathological changes.

### Pharmacokinetics of IBG in a *C. perfringens* infection model

A total of 132 broilers were randomly divided into three groups and a single dose of 2, 30, or 60 mg/kg body weight (b.w.) IBG following oral gavage. At 0.08, 0.25, 0.50, 0.75, 1, 2, 4, 6, 8, 12, and 24 h after oral administration of IBG, four broilers in each group were euthanized to collect ileal contents and blood samples. The concentration of IBG in plasma and ileal content was determined by validated high-performance liquid chromatography (HPLC). In brief, ileal contents (0.5 g) were extracted with 1.5 mL of 1% formic acid acetonitrile, homogenized for 1 min, and centrifuged (13,000 g, 10 min) to obtain supernatant. Subsequently, 0.5 mL of supernatant was added to 1 mL 1% formic acid acetonitrile. After being vortexed (1 min) and centrifuged (13,000 g, 10 min), the supernatant was filtered through a 0.22 μm membrane for concentration analysis. The calibration range was 0.20–20 μg/g. Intraday and interday precision levels varied from 1.1 to 7.2% and from 1.7 to 6.5%, respectively. The LOD and limit of quantification (LOQ) were 0.10 and 0.20 μg/g, respectively. A 0.20 mL aliquot of plasma sample mixed with 0.80 mL of 1% formic acid acetonitrile. After being vortexed (1 min) and centrifuged (13,000 g, 10 min), the supernatant was filtered through a 0.22 μm membrane for concentration analysis. The calibration range was 0.02–1 μg/mL. Intraday and interday precision levels varied from 1.9 to 9.1% and from 2.4 to 8.1%, respectively. The LOD and LOQ were 0.005 and 0.010 μg/mL, respectively. The concentration data of IBG in the plasma and intestinal content were submitted to a non-compartmental analysis in Phoenix WinNonlin^®^ 8.2 (Certara, L.P., Princeton, NJ, USA). The corresponding intestinal content concentration-time profiles after multiple dosage regimens were predicted using Phoenix's non-parametric superposition function based on the single-dose intestinal content PK concentration-time profile.

### Pharmacodynamics of IBG in an intestinal infection model

Infected broilers were treated gavage two times a day for three successive days with 0, 2, 5, 10, 20, 30, 40, and 60 mg/kg b.w. of IBG (*n* = 4) to evaluate the *in vivo* effectiveness of IBG. Treatment started at 12 h post-infection. At 24 h after the last dose, the intestinal content was sampled sterilely and homogenized for CFU determination (26). Broilers in the control group were sacrificed before and 24 h after IBG treatment.

### Analysis of the PK/PD relationship

The *in vivo* PK/PD relationships of IBG in intestinal were simulated using the *I*_*max*_ model in the WinNonlin^®^ 8.2 (Certara, L.P., Princeton, NJ, USA) using the following equation (27):


(1) $$\begin{array}{l} E=E_{0}-\frac{I_{\max}•X}{IC_{50}+X} \end{array}$$

where *E*_0_ is the difference in bacterial count of (log_10_CFU/g) control samples. *I*_*max*_ is the maximum antimicrobial growth inhibition determined as the change in log_10_CFU/g after treatment with IBG. *X* is the predictive variable (AUC_0 − 24*h*_/MIC), and *IC*_50_ is the *X* value producing 50% of the maximum antibacterial effect.

The potential optimal dosage can be calculated using the following equation (28, 29):


(2) $$\begin{array}{l} Dose_{\;}=\frac{\left(AUC/MIC\right)•MIC•CL}{fu•F}, \end{array}$$

where dose (per day) is at a steady state; CL is the clearance per day; AUC/MIC is the targeted endpoint for optimal efficacy in hours; MIC is the target pathogen; F is the bioavailability factor, and *fu* is the free fraction of the drug.

### Cell membrane integrity assay

Cell membrane integrity assay was performed as a previous report (30). *Clostridium perfringens* ATCC13124 were grown overnight at 37°C in an anaerobic system. Then culture cells were washed and resuspended in PBS (pH 7.4) to obtain OD_600_ of 0.5, followed by the addition of 0.5 μmol/L of propidium iodide (PI; Beyotime, Catalog No. ST511) in the presence of IBG (final concentrations ranging from 0 to 16 mg/mL). After incubation for 30 min, fluorescence was measured by using a Hitachi F-7000 Fluorescence Spectrometer with an excitation and emission wavelengths of 535 and 615 nm, respectively.

### Membrane depolarization assay

The membrane potential of cells was using a fluorescent probe DiSC_3_(5) as described previously (14). Then bacterial cells were washed and suspended with 5 mmol/L of HEPES (pH 7.0, plus 5 mM glucose). OD_600_ of bacterial suspension was standardized to 0.5 in the same buffer, and 0.5 μmol/L of 3,3-dipropylthiadicarbocyanine iodide DiSC_3_(5) (Aladdin, Catalog No. D131315) was added. After incubation at 37°C for 30 min, 190 μL of probe-labeled bacterial cells was added to a 96-well plate and 10 μL of IBG (final concentrations ranging from 0 to 16 mg/mL) was added. After incubation at 37°C for 30 min, fluorescence was measured with an excitation wavelength at 622 nm and an emission wavelength at 670 nm.

### Proton motive force assay

The PMF of *C. perfringens* ATCC13124 treated with IBG was measured with pH-sensitive fluorescence probe BCECF-AM (20 × 10^−6^ M, UElandy Catalog No. B3016). After the fluorescence was stabilized, varying IBG were added. The excitation and emission wavelengths on the fluorescence spectrometer were set to 488 and 525 nm, respectively.

### ATP determination

Intracellular ATP levels of *C. perfringens* ATCC13124 were determined using an Enhanced ATP Assay Kit (Beyotime, Catalog No. 50027). *C. perfringens* ATCC13124 grown overnight at 37°C in an anaerobic system was washed and resuspended to obtain OD_600_ of 0.5 with PBS (pH 7.4). After treating with different concentrations (0–16 mg/L) of IBG for 30 min, bacterial cultures were centrifuged and the supernatant was removed. Bacterial precipitates were lysed with lysozyme and centrifuged, and the supernatant was prepared for measurement at intracellular ATP levels. Recording in the luminescence model using the Hitachi F-7000 Fluorescence Spectrometer.

## Results

### *In vitro* susceptibility testing and time-killing assays

**Figure 1:**
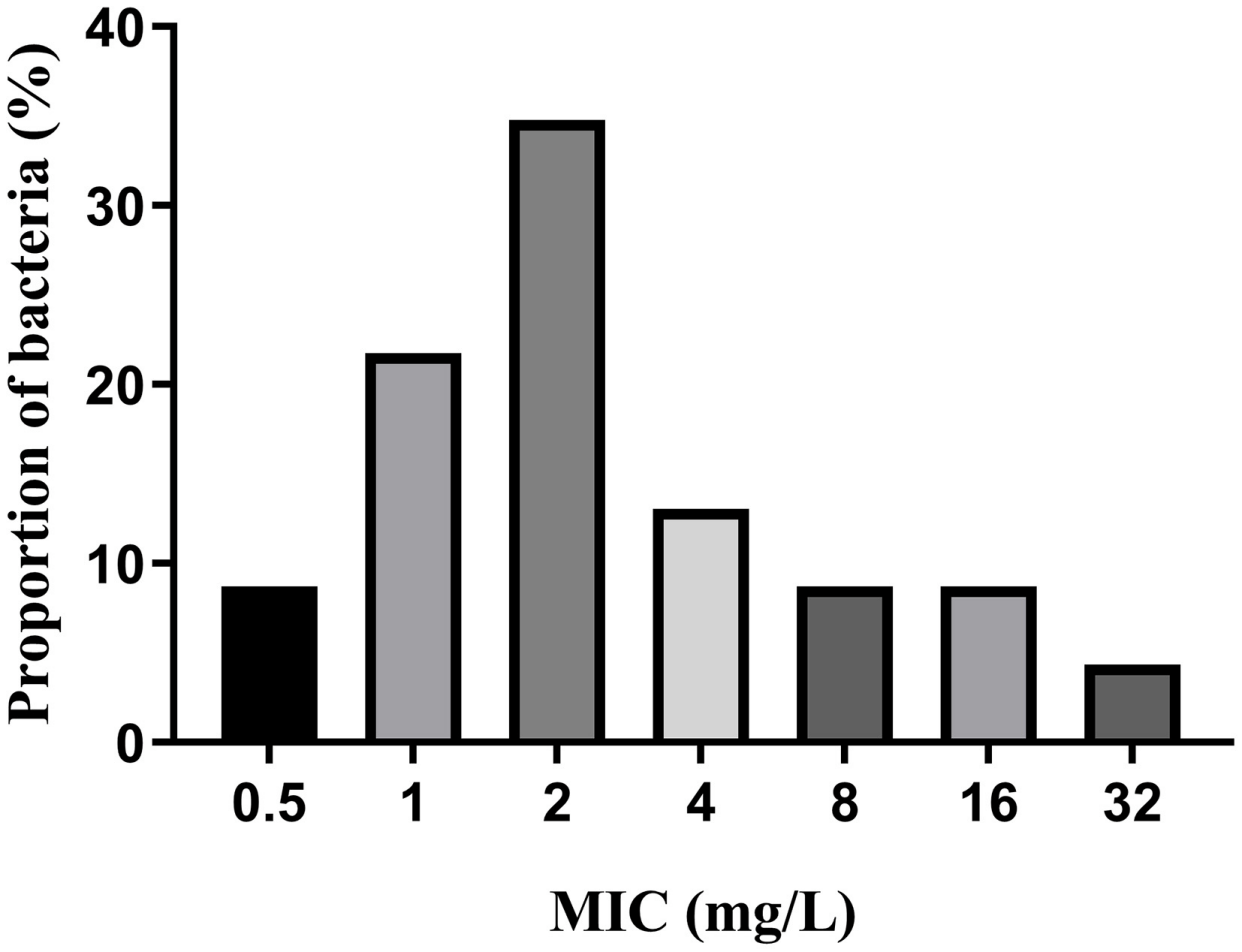
MIC distributions of IBG against 23 *C. perfringens*.

**Figure 2:**
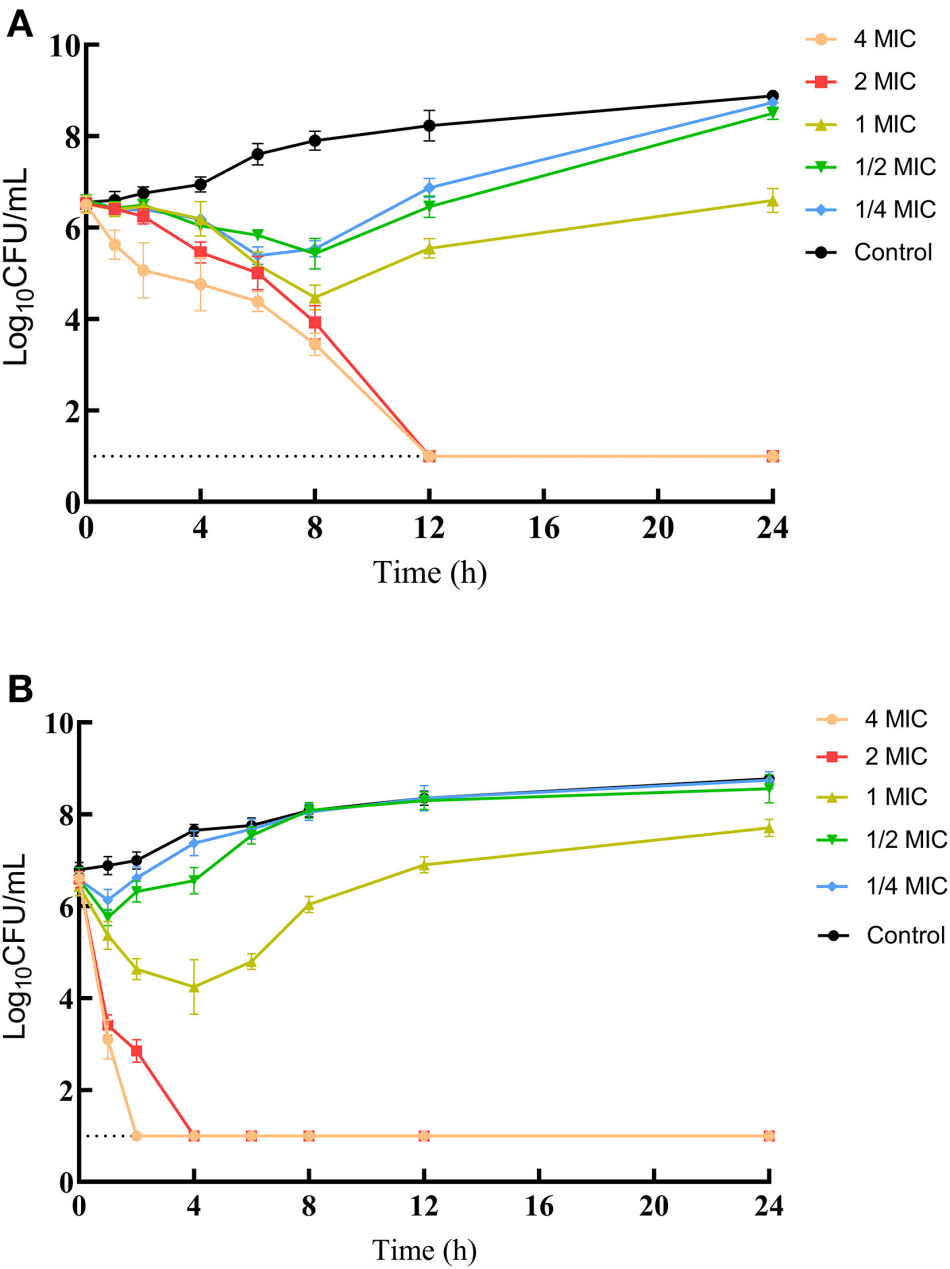
*In vitro* time-kill curve of IBG against *C. perfringens* ATCC13124 **(A)** and GDZ21C59W **(B)**.

MICs of IBG against 23 *C. perfringens* strains varied, ranging from 0.5 to 32 mg/L. The percentage of each MIC (0.5, 1, 2, 4, 8, 16, and 32 mg/L) was 8.70, 21.74, 34.78, 13.04, 8.70, 8.70, and 4.35%, respectively. The MIC distribution is shown in Figure 1. The MIC and MBC of IBG against *C. perfringens* ATCC13124 in MH broth were 2 and 4 mg/L, whereas those in ileal content were eight times higher at 16 and 32 mg/L, respectively. The MPC in the medium was eight times higher than the MIC, with a value of 16 mg/L. The PAE of *C. perfringens* exposed to IBG for 1 and 2 h ranged from 0.39 to 1.37 h and from 0.82 to 1.51 h, respectively (Table 1). The *in vitro* time-killing curves of IBG against *C. perfringens* ATCC13124 and GDZ21C59W in the MH broth are illustrated in Figure 2. The time-killing curves imply a concentration–dependent killing characteristic of IBG. When *C. perfringens* was exposed to IBG with a concentration >2 mg/L, the continuous inhibitory effect on bacterial growth could be observed.

**Table 1:** Antibacterial activity of IBG against *C. perfringens* ATCC13124.

	**MIC (mg/L)**	**MBC (mg/L)**	**MPC (mg/L)**	**PAE (h)**		
				**Concentration**	**Expose 1 h**	**Expose 2 h**
Artificial medium	2	4	16	1 MIC	0.39	0.82
				2 MIC	0.85	0.92
				4 MIC	1.37	1.51
Ileal content	16	32	–	–	–	–

### Pharmacokinetics analysis

**Figure 3:**
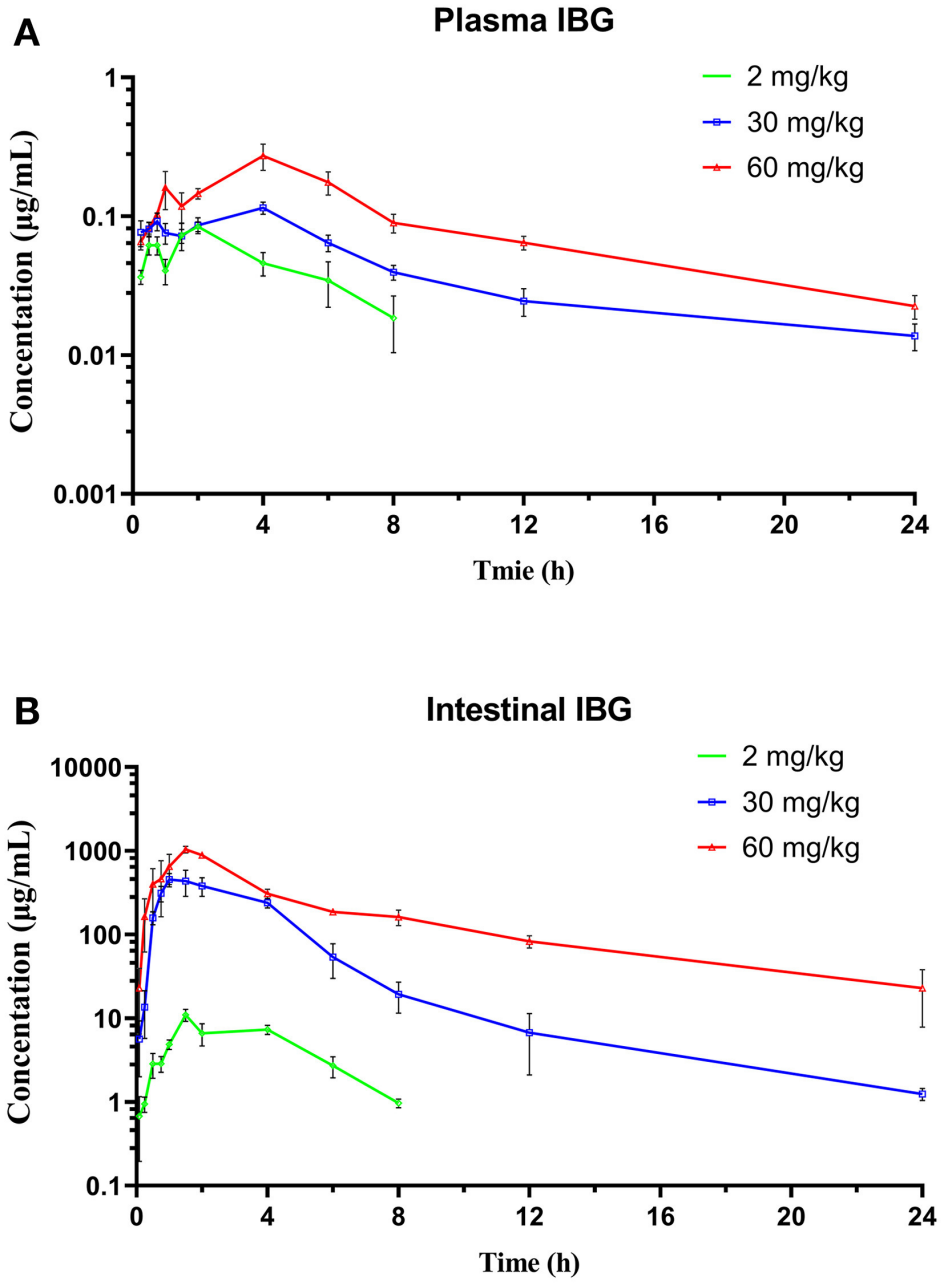
The time–concentration profile of IBG in plasma **(A)** and intestinal contents **(B)** of broilers following a single oral administration of 2, 30, and 60 mg/kg (*n* = 4).

The concentration–time profiles of plasma and intestinal content in *C. perfringens*-infected broilers following single oral gavage at 2, 30, and 60 mg/kg is shown in Figure 3. The PK parameters of IBG in plasma and intestinal content are illustrated in Table 2. After oral administration, IBG had a significantly lower AUC_last_ and C_max_ in plasma vs. in intestinal content (*P* < *0.01*). In plasma, AUC_last_ and C_max_ ranged from 0.38 to 2.18 mg·h/L and from 0.08 to 0.27 mg/L, respectively. In intestinal content, AUC_last_ and C_max_ ranged from 38.31 to 4,266.77 mg·h/L and from 10.97 to 1,036.64 mg/L, respectively. A good linearity of IBG was observed in the intestine (*R*^2^ ≥ 0.988 for C_max_ and AUC_last_).

**Table 2:** Pharmacokinetic parameters of IBG in plasma and ileal content following single gavage in *C. perfringens*-infected broilers.

**Dose (mg/kg)**	**Plasma**				**Ileal content**			
	***T_*max*_* (h)**	***C_*max*_***	***AUC_*last*_***	***T_1/2_* (h)**	***T_*max*_* (h)**	***C_*max*_***	***AUC_*last*_***	***T_1/2_* (h)**
2	2	0.08	0.38	4.06	1.50	10.97	38.31	2.04
30	4	0.12	0.99	11.21	1.00	452.70	1,688.93	4.22
60	4	0.27	2.18	7.99	1.50	1,036.64	4,266.77	5.83
Mean ± SD	3.33 ± 0.94	–	–	7.75 ± 2.92	1.33 ± 0.24	–	–	4.03 ± 1.55

T_max_, time of maximum observed concentration; C_max_, maximum concentration; AUC_last_, the area under the concentration–time curve from 0 h to the last sample time point; T_1/2_, half-life.

### PK/PD analysis

**Figure 4:**
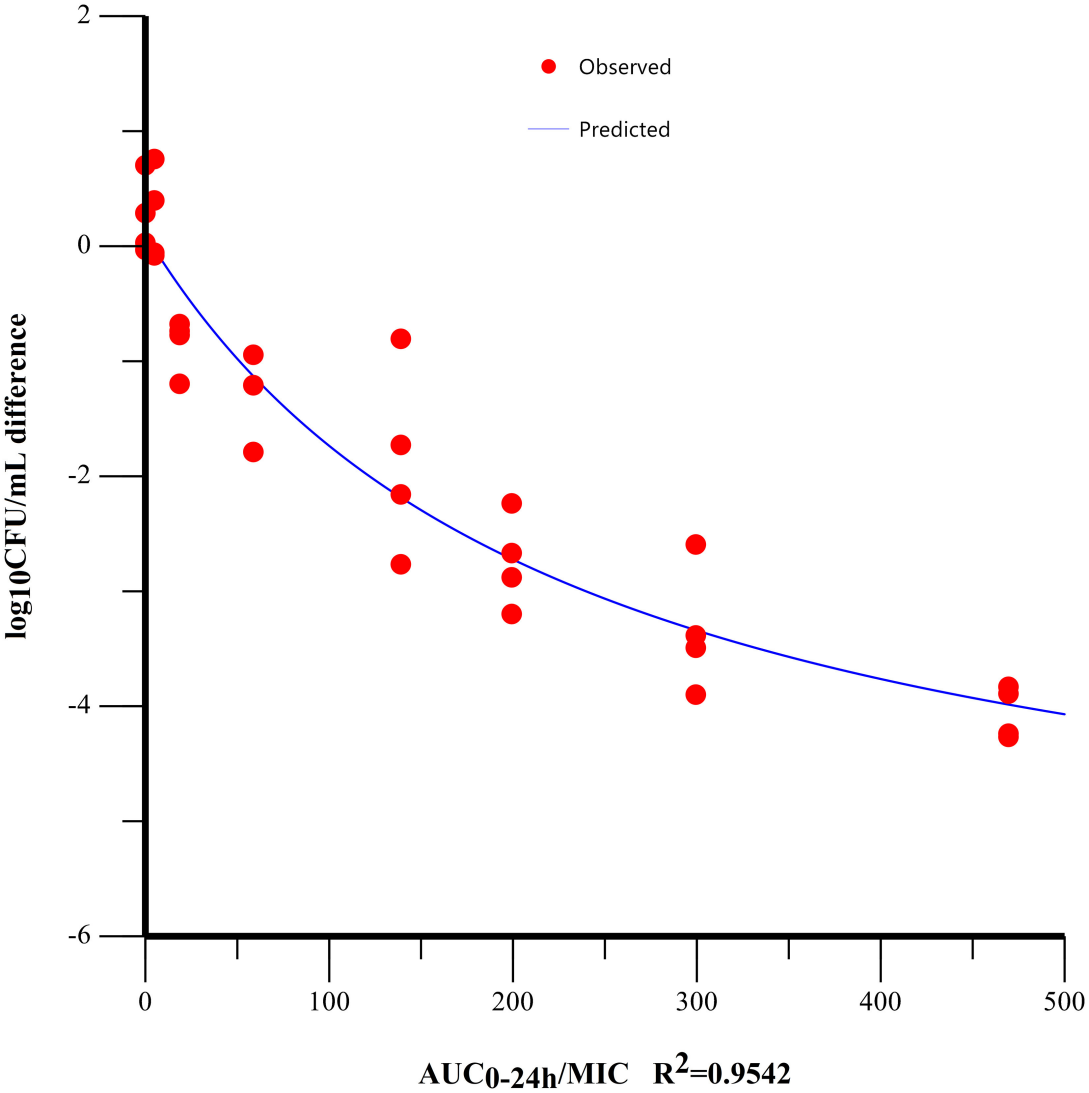
Relationships between the effect of IBG against *C. perfringens* and PK/PD indices AUC_0 − 24*h*_/MIC in the intestinal infection model. *R*^2^ is the coefficient of determination.

At the start of IBG therapy, bacterial burdens were 8.05 ± 0.25 log_10_CFU/g. The most effective IBG dosage regimens result in the reduction of bacterial number at the start of treatment (4.06 ± 0.19 log_10_CFU/g). The relationship between the effect of IBG against *C. perfringens* and each of the PK/PD indices in the intestinal infection model is shown in Figure 4. The PK/PD index of AUC_0 − 24*h*_/MIC (*R*^2^ > 0.9542) had a strong correlation with antibacterial activity in the intestinal infection model. The AUC_0 − 24*h*_/MIC ratios required for various efficacy targets are shown in Table 3.

**Table 3:** PK/PD parameter of *in vivo* data after oral administration IBG in broilers.

**Parameter**	**Unit**	**PK/PD fitting parameters**
*E_0_*	(log_10_CFU/g)	0.11
*I_*max*_*	(log_10_CFU/g)	6.11
*IC_50_*	h	232.03
AUC_0 − 24*h*_/MIC for bacteriostatic action	h	4.00
AUC_0 − 24*h*_/MIC for bactericidal action	h	240.74
AUC_0 − 24*h*_/MIC for bacterial elimination	h	476.98

E_0_, difference in number of bacteria counts (log_10_ CFU/g) in a drug-free sample between 0 and 24 h; I_max_, difference in greatest amount of antibacterial reduction (log_10_ CFU/g); IC_50_ is the AUC_0 − 24*h*_/MIC value producing 50% of the maximal antibacterial effect.

### IBG disrupted cell membrane in multiple ways

**Figure 5:**
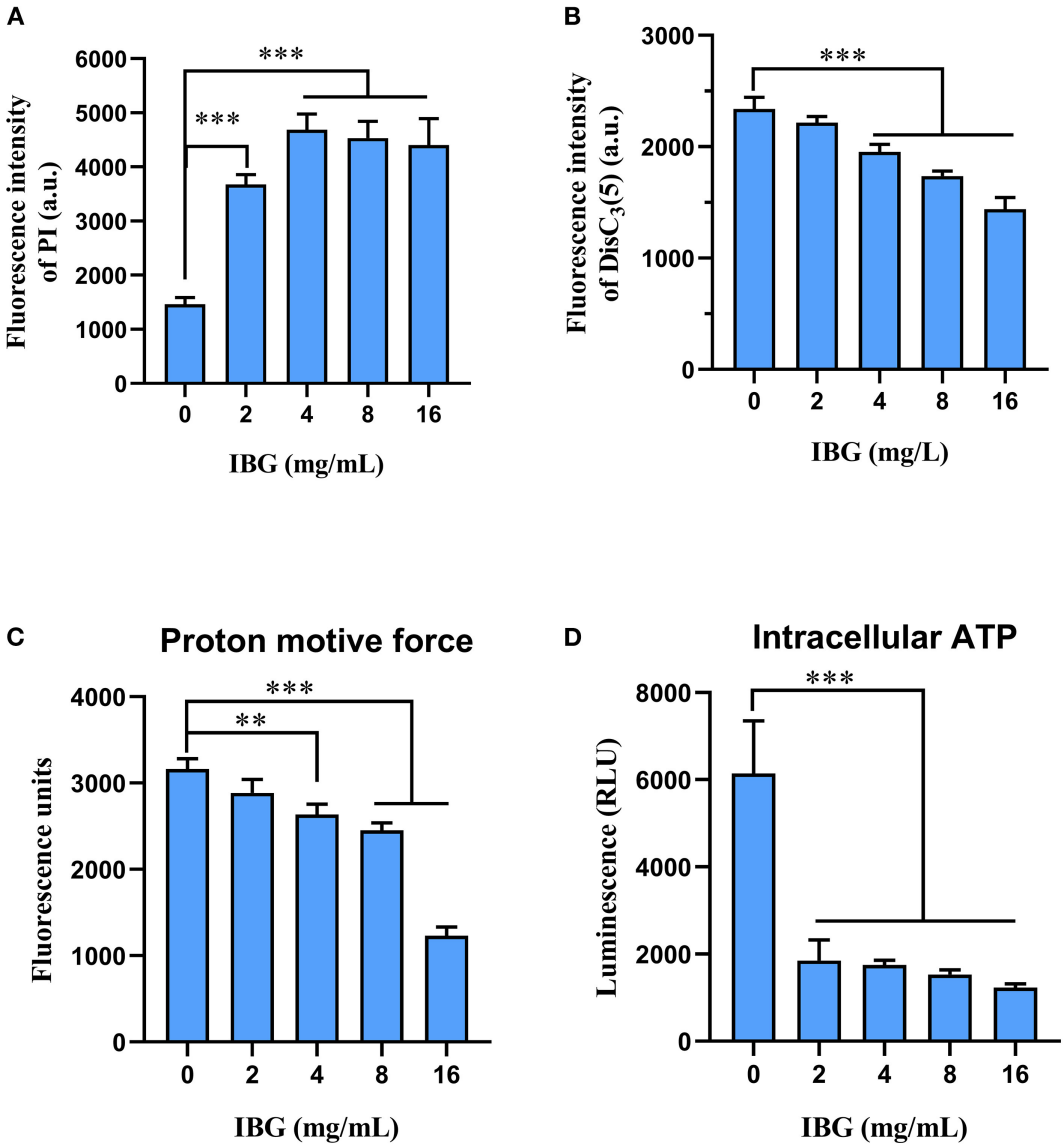
Mechanism of IBG against *C. perfringens*. **(A)** Increased permeability of the inner membrane of *C. perfringens* ATCC13124 treated with IBG (0–16 mg/L) for 30 min. **(B)** IBG dissipates membrane potential of *C. perfringens* ATCC13124. **(C)** Disruption of PMF with increased IBG by monitoring the fluorescence intensity of BCECF-AM-probed *C. perfringens* cells. **(D)** Decreased levels of intracellular ATP in *C. perfringens* ATCC13124 after treatment with IBG. All data are presented as mean ± SD, and the significant difference was determined by non-parametric one-way ANOVA (**p < 0.01, ***p < 0.001).

Based on IBG killing against *C. perfringens in vitro* and *in vivo*, we try to elucidate its potential mechanisms. In addition, IBG killing against *Staphylococcus aureus* and *Enterococcus* by damaging cell membrane (16, 17), may exert antibacterial activity against *C. perfringens* in a similar manner. Thus, we first tested the effect of IBG on the permeability of the cytoplasmic membrane. We used a fluorescent probe PI to assess the effect of IBG on the inner membrane of the bacteria as previously described (31). The results showed that IBG increased the permeability of *C. perfringens* ATCC13124 (Figure 5A). The fluorescence value clearly increased with IBG treatment compared with that of untreated cells., which indicated that IBG might cause dysfunctions in the cytoplasmic membrane. Hence, DiSC_3_(5) was used to evaluate the bacterial membrane potential (32). When the concentration of IBG was more than four times that of MIC, the fluorescence was significantly reduced, suggesting that IBG disrupted the electric potential of *C. perfringens* (Figure 5B). Existing research has shown that membrane depolarization is related to the production of ROS and PMF (33, 34). Dyes DCFH-DA (35) and BCECF-AM (36) were used to analyze the effects of ROS and PMF, respectively. There was no effect on ROS accumulation in *C. perfringens* treated with IBG. A large reduction in the magnitude of PMF accumulation was observed in the IBG-treated group compared with untreated cells (Figure 5C). Considering that PMF is the driving force for ATP synthesis (37), the intracellular ATP levels of *C. perfringens* treated with IBG was also significantly decreased (Figure 5D). Collectively, IBG stimulates a membrane-dependent mechanism to exert an antibacterial effect.

## Discussion

Due to overuse and misuse, and resistance to commonly used antibiotics, the control of *C. perfringens* is very difficult (38). Therefore, new antimicrobial drugs are needed for the effective management of necrotizing enteritis. IBG, as a novel guanidine substituted compound, has been shown to be effective against Gram-positive bacteria by disrupting cell membrane (16, 17). In this study, the MIC range of IBG to clinical *C. perfringens* strains was 0.5–32 mg/L. Given the differences in bacterial growth under *in vitro* and *in vivo* conditions, the MIC in MH broth and ileal content were detected in this study. The MIC of IBG against *C. perfringens* ATCC13124 in an MH broth and ileal content was 2 and 16 mg/L, respectively. *In vitro* antibacterial effects of IBG under different conditions were quite different. Therefore, using the MIC in ileal content is more appropriate when calculating the PK/PD index of AUC_0 − 24*h*_/MIC.

Based on the PK results, the absorption and distribution of IBG in the intestine of broilers were rapid following oral gavage. Therefore, IBG can be used to treat intestinal bacterial infections. Previous PK/PD studies mostly focused on the integration of PK and the serum or plasma of PD parameters to indicate the relationship between drug time-course and curative effect (39, 40). Taking the intestine as the target organ, the plasma concentration of oral non-absorbable drugs such as colistin is negligible, which cannot provide an effective quantification of the gastrointestinal antibacterial effect (41). In this investigation, we described the PK of IBG in *C. perfringens*-infected broiler ileal content for PK/PD investigations. The concentration of IBG in ileal content increased gradually after gavage, and then decreased rapidly with the transportation of chyme, which was similar to the pharmacokinetic characteristics of other oral non-absorbable drugs (24). Thus, we used intragastric administration once every 12 h for *in vivo* PD study.

PK-PD analysis has become an important tool for formulating rational dosage regimens and preventing the emergence of antimicrobial drug resistance (42). In this study, the PK/PD index of AUC_0 − 24*h*_/MIC (*R*^2^ > 0.9542) had a strong correlation with antibacterial activity in the intestinal infection model. The AUC_0 − 24*h*_/MIC targets required to achieve bacteriostatic, bactericidal, and virtual eradication effect were 4.00, 240.74, and 476.98 h, respectively. According to the dosage equation, the dose regimen could be calculated. In this study, the MIC of *C. perfringens* isolate was 2 mg/L. For dosage calculation, bioavailability considered because of the extravascular route of administration, and Cl/F in ileal content was 0.03 ± 0.02 L/kg·h. *fu* was not required for using PD data generated in the small intestine (20). For the treatment of *C. perfringens* with MIC ≤ 2 mg/L, the dose of IBG for therapeutic and elimination of *C. perfringens* was 12.98 and 25.71 mg/kg repeated every 12 h, respectively.

This study was the first to demonstrate the antibacterial activity of IBG against *C. perfringens in vitro* and *in vivo* and then determine the AUC_0 − 24*h*_/MIC targets in the intestine of broilers, which were simulated using an *Imax* model. In addition, IBG displayed a potent antimicrobial activity against *C. perfringens* by targeting the cell membrane. The results demonstrate that IBG has the promising potential to become a new class of antimicrobials for the treatment of *C. perfringens* infections in broilers.

## Data availability statement

The original contributions presented in the study are included in the article/supplementary material, further inquiries can be directed to the corresponding authors.

## Ethics statement

The animal study was reviewed and approved by Institutional Animal Care and Use Committee of South China Agricultural University.

## Author contributions

DZ and ZZ conceived this study and participated in its design and coordination. YL and LY designed the experiments and drafted the manuscript. YL, LY, and WZ carried out the *in vivo* animal experiments. YL, LY, and JL carried out the mechanism experiments. XP and ZQ worked on the synthesis of compound IBG. DZ, ZZ, and YL conducted the PK/PD analysis. All authors read and approved the final manuscript.

## Funding

This work was supported by the Foundation for Innovative Research Groups of the National Natural Science Foundation of China (Grant No. 32121004).

## Conflict of interest

Authors XP and ZQ were employed by Guangzhou Insighter Biotechnology Co., Ltd. The remaining authors declare that the research was conducted in the absence of any commercial or financial relationships that could be construed as a potential conflict of interest.

## Publisher's note

All claims expressed in this article are solely those of the authors and do not necessarily represent those of their affiliated organizations, or those of the publisher, the editors and the reviewers. Any product that may be evaluated in this article, or claim that may be made by its manufacturer, is not guaranteed or endorsed by the publisher.
